# Evaluation of *Centella asiatica* Germplasm Resources in Yunnan, China, Based on Phenotypic Traits, SSR Markers, and Chemical Profiling

**DOI:** 10.3390/plants15152345

**Published:** 2026-07-30

**Authors:** Fan Yang, Qixiang You, Yanbing Ma, Zhenghai Sun, Liping Li, Weiwei Li

**Affiliations:** 1 Yunnan International Joint R&D Center for Integrated Utilization of Ornamental Grass, Yunnan Key Laboratory of Landscape Plant Resource Cultivation and Application, College of Landscape and Horticulture, Southwest Forestry University, Kunming 650224, China; yf210407@163.com (F.Y.); yqx018262020@outlook.com (Q.Y.); mayanbing@swfu.edu.cn (Y.M.); 2 College of Ecology and Environment (College of Wetlands), Southwest Forestry University, Kunming 650224, China; 3 Yunnan Key Laboratory of Biodiversity Information, Kunming Institute of Zoology, Chinese Academy of Sciences, Kunming 650223, China; kmlww@163.com

**Keywords:** *Centella asiatica*, germplasm resources, phenotypic traits, SSR markers, chemical compounds, high-performance liquid chromatography

## Abstract

*Centella asiatica* (L.) Urb., a perennial herb of the *Apiaceae* family, is an important medicinal plant with diverse therapeutic applications. In this study, we integrated phenotypic evaluation, SSR markers, and HPLC fingerprinting to assess the diversity of 24 *C. asiatica* accessions collected from Yunnan Province, China. Considerable phenotypic, genetic, and biochemical diversity was observed. The Shannon–Wiener index ranged from 2.20 to 3.14 for qualitative traits. Principal component analysis identified five components accounting for 82.54% of the total phenotypic variance, with leaf morphology as the primary determinant. Q-type cluster analysis grouped the accessions into five phenotypic clusters. Transcriptome analysis revealed 16,799 SSR loci, with dinucleotide repeats as the most frequent motif (44.31%). UPGMA clustering based on SSR markers delineated five genetic groups that correlated with geographic origin, with accessions from the two northernmost sites forming a distinct cluster. HPLC fingerprints identified 16 common peaks, among which five major bioactive constituents—madecassoside, asiaticoside, kaempferol, madecassic acid, and asiatic acid—were quantified. The total content of these five compounds varied markedly among accessions (4.09–20.18 mg·g^−1^), with the highest found in Zhaoyang (S5). Most accessions exhibited high chemical similarity (>0.9), except for Yiliang (S12), which displayed a distinct profile. The partial correlation between SSR-based classifications and geographic origin, together with the inconsistency between phenotypic and chemical groupings, indicates that morphological variation is shaped by both environmental factors and complex genetic regulatory mechanisms. These findings provide a scientific basis for germplasm conservation, selective breeding, and the sustainable utilization of *C. asiatica*.

## 1. Introduction

*Centella asiatica* (L.) Urban is a perennial creeping herb belonging to the genus *Centella* in the Apiaceae family. It is found in tropical and subtropical regions stretching from Asia and Africa to Australia; in particular, it is widely distributed in the provinces and regions south of the Yangtze River in China. In traditional Asian medicine, *C. asiatica* has been used medicinally for over a thousand years. The Divine Farmer’s Materia Medica (Shennong Bencao Jing, the earliest classic of Chinese materia medica) classifies it as a “medium-grade” herb, noting its functions in clearing heat, draining dampness, detoxifying, and reducing swelling. The main active components of this plant belong to the pentacyclic triterpenes class, including madecassoside, asiaticoside, madecassic acid, and asiatic acid [1]. These substances have demonstrated considerable effectiveness in anti-inflammatory actions, wound healing, anti-fibrotic properties, and neuroprotection [2,3,4,5], thus increasing attention on *C. asiatica* in the field of pharmaceuticals and functional products.

From an agricultural perspective, *C. asiatica* is not only a medicinal plant of economic value but also a valuable model for investigating the biosynthesis of plant triterpenoid saponin synthesis. Recently, several cytochrome P450 (CYP) genes involved in triterpene biosynthesis have been cloned [6,7]. Additionally, Asiatic acid has been successfully synthesized de novo in the yeast *Saccharomyces cerevisiae* [8,9]. Regarding cultivation, the effects of environmental factors such as photoperiod, light-dark cycles, and salt stress on the accumulation of *Centella saponins* have been increasingly clarified [10,11,12], and studies under hydroponic conditions have laid the groundwork for future large-scale production [13]. Nevertheless, the industrial utilization of *C. asiatica* remains constrained by insufficient knowledge of the available genetic resources. In particular, a comprehensive evaluation system integrating the morphological, genetic, and chemical diversity of accessions from different geographic origins is still lacking. This has thus hindered the selection of superior genetic resources and the directed breeding of new varieties.

Regarding the morphological and phenotypic variations, Ali et al. [14] did a comparative study. They examined Australian *C. asiatica* and two closely related species, which are quite similar to it, *C. cordifolia* and *C. erecta*, and found that *C. asiatica* has smaller leaves with less pronounced serrations on the margins, thin, and soft stems growing prostrate on the ground, 6 to 8 vascular bundles visible in the cross-section, and a periderm in the older stems that contains red anthocyanins. In contrast, the two closely related species have serrated leaf margins, thick and stiff stems, and grow more like small shrubs. They believe that the combination of leaf morphology and stem growth habit is important for distinguishing these species. Even within the same species, *C. asiatica* from different places usually differ in flowering and fruiting times as well as plant height. Sometimes, these differences persist even after transplanting to the same plot of land, suggesting that genetic changes may have occurred as the plant adapts to different native environments over time [14]. There are also differences in chemical composition depending on geographic origin. Puttarak et al. [15] found that leaves contain the highest levels of pentacyclic triterpenes, and that content varies by geographic origin and harvest season. Pinit et al. [12] applied metabolomics to study *C. asiatica* with different genotypes in Thailand. Their results showed that even for a single factor, such as salt stress, its promoting effect on secondary metabolite accumulation depends on the specific genotype. This indicates that the ability of a certain variety to produce high-quality medicinal materials under certain environmental conditions is due to the interaction between genetics and the environment. Moreover, Chysirichote and Aroonsong [16] developed a stepwise processing method combining pH regulation with enzymes to selectively release the glycosides of *Centella*, thereby identifying more appropriate processing methods for materials of different chemical types.

Effective management of germplasm resources needs a molecular analysis of genetic diversity. The first systematic study was done by Rakotondralambo’s team, who isolated microsatellite markers from an enriched genomic library and then examined wild populations of *C. asiatica* in Madagascar. They discovered an average of 4.3 alleles per locus and identified numerous unique alleles across morphotypes. In that study, *C. asiatica* was reported to be diploid (2*n* = 18), and previous controversies over chromosome number, B chromosomes, and ploidy were discussed [17]. Subsequently, researchers from various regions of the world conducted genetic structure and diversity analyses of *C. asiatica* populations across different areas using simple sequence repeat (SSR) and inter-simple sequence repeat (ISSR) markers, finding that most genetic variation occurs among individuals within populations, with both inbreeding and population differentiation acting simultaneously [18,19,20,21]. Deng et al. [20] recently utilized transcriptomic data to develop a large set of EST-SSR markers and observed genetic differentiation in terpenoid biosynthesis-related genes among populations. Wan et al. [22] combined metabolomic and transcriptomic data for analysis, identifying several candidate genes in *C. asiatica* leaves that may be involved in triterpene saponin biosynthesis, thereby providing additional targets for future genetic improvement.

Chemical composition analysis and quality assessment revealed significant differences in the chemical composition among various *C. asiatica* accessions. Li, J. et al. [23] analyzed the chemical profiles of roots, stems, and leaves using liquid chromatography–mass spectrometry (LC-MS), and found that triterpenoid glycosides were mainly distributed in the leaves, while the proportion of aglycones was higher in the roots, each organ having its own preference for accumulation. Li, Y. et al. [24] not only measured the content of three major triterpenoid saponins but also correlated these levels with antibacterial activity. Methodologically, Yang, R. et al. [25] optimized the analytical methods for major triterpenoids, while Dang’s team [26] developed an offline three-dimensional chromatography coupled with high-resolution ion-current mass spectrometry (IC-MS) technique, identifying over 200 compounds from *C. asiatica* in a single run. Maliwong et al. [27] not only optimized the HPLC-UV analytical conditions but also identified new polyacetylene glucoside components. Fingerprinting techniques have also been used to evaluate the quality consistency of *C. asiatica* from different origins. For instance, Li et al. established a UPLC fingerprint method combined with Principal Component Analysis (PCA) and cluster analysis, which successfully discriminated *C. asiatica* samples from various geographical regions, demonstrating high precision, stability, and repeatability [28]. Moreover, several new methods have been tested to efficiently extract active ingredients, such as combining cold plasma pretreatment with eutectic solvents [29] or microwave-assisted extraction [30], which can greatly enhance the extraction efficiency.

Yunnan, a region in southwestern China, is a special area: a low-latitude plateau with varied topography and distinct climate zones, famous for its rich biodiversity. It is called the “Kingdom of Flora and Fauna” [31,32]. Many regions with high genetic diversity of important medicinal herbs are situated in Yunnan. However, to date, systematic studies on the germplasm resources of *C. asiatica* in Yunnan have not been conducted. Therefore, it is difficult to address important problems, such as the extent of *C. asiatica* resources in Yunnan, their spatial distribution, and which germplasm has the best comprehensive quality. Fortunately, in recent years, some methods that integrate phenotypic traits with molecular markers [33] or use High-Performance Liquid Chromatography (HPLC) fingerprinting together with chemometrics for evaluation [34] have proved efficient in assessing the resources of several medicinal plants, providing us with both techniques and confidence.

Based on this background and these questions, we collected 24 accessions of *C. asiatica* from different sites in Yunnan Province to carry out a comprehensive evaluation of these resources at three levels. First, we carefully observe and record the phenotypic traits, including stems, leaves, flowers, and fruits, and then analyze the data using multivariate statistical techniques to evaluate the total phenotypic diversity and to identify the main factors affecting it. Secondly, we apply SSR molecular markers to study the genetic diversity and genetic structure of these materials in order to elucidate the phylogenetic relationships among germplasm from various regions and to investigate the correlation between these relationships and geographical distribution. Furthermore, we will construct HPLC chemical fingerprint patterns, detect the common peaks, and compare the quantities of the five major bioactive substances: madecassoside, asiaticoside, kaempferol, madecassic acid, and asiatic acid. The objective is to integrate and analyze these diverse datasets to provide a robust foundation for identifying superior *Centella asiatica* germplasm from Yunnan, supporting conservation strategies, and advancing selective breeding and practical applications.

## 2. Results

### 2.1. Analysis of Phenotypic Trait Diversity

To assess the extent of phenotypic diversity among the collected germplasm, we first characterized 17 morphological traits (12 quantitative and 5 qualitative) across the 24 accessions. The 24 accessions of *C. asiatica* were collected from Yunnan Province, and the descriptive statistics and diversity indices are presented in Table A5 and Table A6. Among the 12 quantitative traits, the coefficients of variation (CV) range from 8.8% to 48.4%, with an average of 22.96%. The Shannon-Wiener diversity index (H’) varies from 2.64 to 3.17 across the quantitative traits, suggesting a relatively high level of diversity among the analyzed accessions. Among these traits, petiole length (LPL) exhibits the highest variability (CV = 48.4%), varying from 2.17 cm to 14.40 cm, followed by leaf area (LA) (CV = 46.7%). Fruit width (FW) has the lowest variability (CV = 8.8%), with values from 2.06 mm to 3.77 mm. The five qualitative traits were classified into two phenotypic categories each (Table A6). The Shannon–Wiener diversity index (H’) for these qualitative traits ranged from 2.20 to 3.14. Among them, stem node color showed the most balanced distribution, with 62.5% of accessions exhibiting light purple nodes and 37.5% showing purplish-red nodes (H’ = 2.20). In contrast, flower color exhibited the most skewed distribution, with purplish-red flowers accounting for 95.83% of the samples and milky-white flowers observed in only one accession (4.17%).

### 2.2. Correlation Analysis of Phenotypic Traits in C. asiatica

The correlation analysis of 17 phenotypic traits in 24 *C. asiatica* accessions is presented in Table A7. Strong positive correlations were consistently observed among leaf size-related traits. Specifically, leaf area (LA) was highly correlated with leaf length (LL, *r* = 0.972, *p* < 0.01), leaf width (LW, *r* = 0.980, *p* < 0.01), petiole length (LPL, *r* = 0.855, *p* < 0.01), and petiole diameter (LPD, *r* = 0.834, *p* < 0.01). These four traits were also strongly intercorrelated (all pairwise r > 0.76, *p* < 0.01). Additionally, leaf size traits showed significant positive correlations with the number of marginal teeth (NLS), leaf surface wrinkling (WLSW), fruit length (FL), and internode diameter (SD) (r = 0.475–0.698, *p* < 0.05 or *p* < 0.01). Wider leaves generally exhibited a less elongated shape, as reflected by the negative correlation between LW and the length-to-width ratio (LL/LW; *r* = −0.455, *p* < 0.05). Among all trait pairs, the highest correlation coefficient was observed between LA and LW (*r =* 0.980, *p <* 0.01), followed by LA and LL (*r =* 0.972, *p <* 0.01) and LL and LW (*r =* 0.930, *p <* 0.01). These results indicated that leaf morphology represents the most tightly integrated component of phenotypic variation within the analyzed *C. asiatica* germplasm. By contrast, stem node color (SC) exhibited significant or highly significant negative correlations with LA, LPL, LPD, LL, LW, NLS, LS, and WLSW, indicating that accessions with purplish-red nodes (SC = 1) tended to have smaller and smoother leaves.

### 2.3. PCA and Cluster Analysis of Phenotypic Traits in C. asiatica

**Figure 1:**
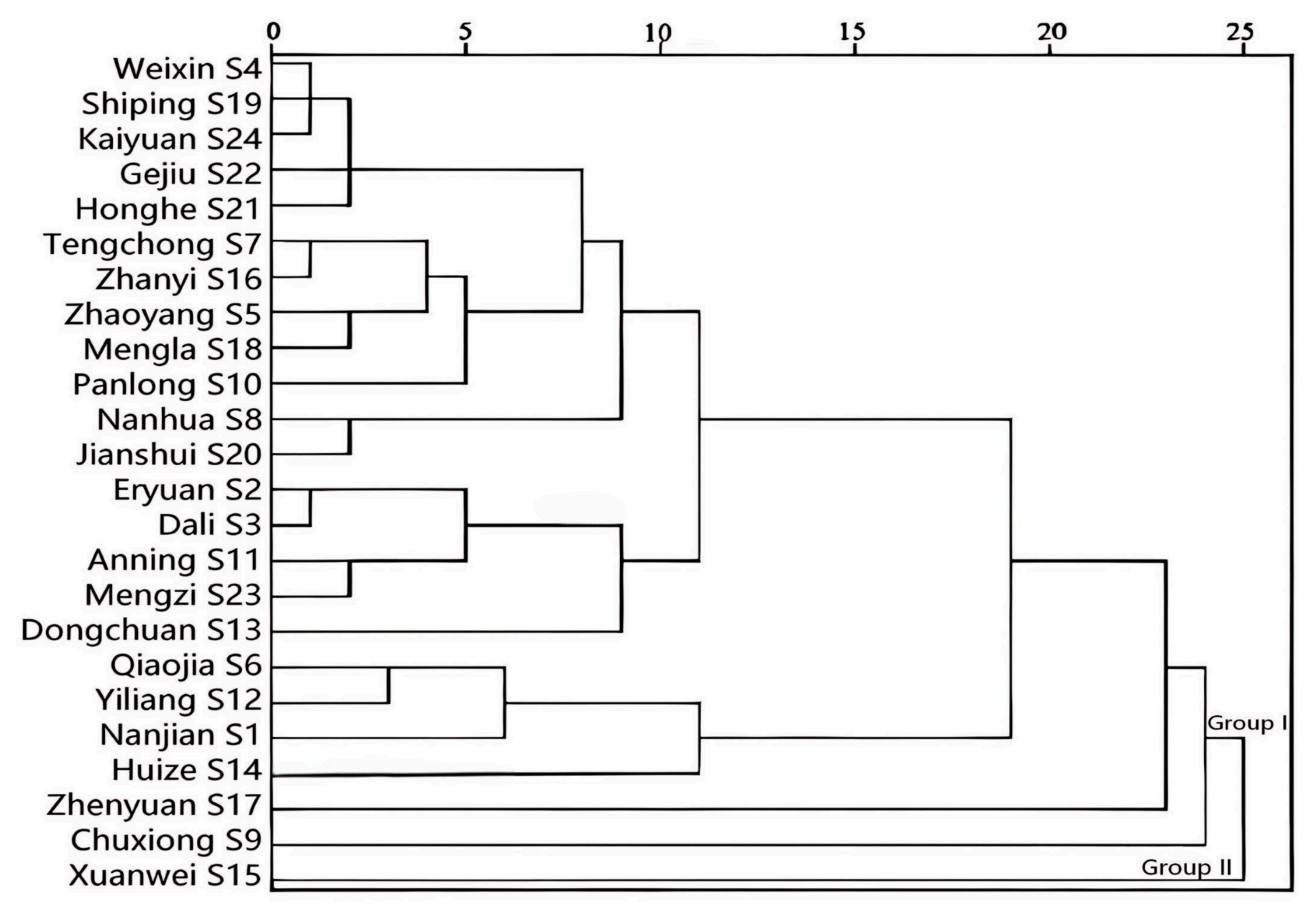
Q-clustering analysis of 24 samples of *C. asiatica* from different regions in Yunnan Province.

**Figure A1:**
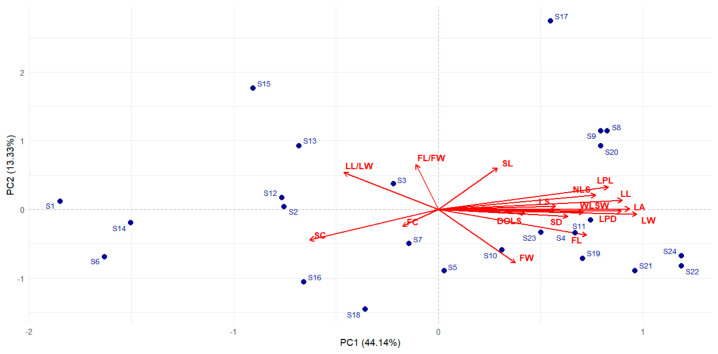
PCA biplot of 17 phenotypic traits and 24 *C. asiatica* accessions based on the first two principal components. Red arrows represent the loadings of individual traits on PC1 and PC2, with arrow length proportional to the contribution of each trait. Blue points indicate the scores of the 24 accessions. PC1 and PC2 together account for 57.47% of the total phenotypic variation.

PCA was performed using the 17 phenotypic traits recorded for the 24 *C. asiatica* accessions. The first five principal components had eigenvalues greater than 1 and together accounted for 82.54% of the total phenotypic variation (Table A8 and Table A9), indicating that these components adequately summarized most of the morphological diversity within the germplasm collection. A PCA biplot displaying the loadings of the 17 phenotypic traits and the scores of the 24 accessions along the first two principal components is provided in Figure A1. The first principal component had an eigenvalue of 7.504 and contributed 44.14% of the total variation. PC1 mainly reflected leaf size and vegetative development, as indicated by the high loadings of leaf width (LW, 0.970), leaf area (LA, 0.934), leaf length (LL, 0.898), petiole diameter (LPD, 0.893), and petiole length (LPL, 0.830) (Table A8). The eigenvalue and contribution rate of the second principal component were 2.266 and 13.33%, respectively, and this component was mainly associated with fruit length-to-width ratio (FL/FW) and internode length (SL). Based on the eigenvalues and contribution rates, leaf-related morphological traits, fruit length-to-width ratio, and internode length were identified as the main determinants of phenotypic variation in *C. asiatica*, with flower color contributing primarily to the fifth principal component. Since leaf area (LA) was highly correlated with leaf width (LW, *r* = 0.980), leaf length (LL, *r* = 0.972), petiole length (LPL, *r* = 0.855), and petiole diameter (LPD, *r* = 0.834), and these four traits were also strongly correlated with each other (all pairwise r > 0.76), they were excluded from the subsequent Q-type cluster analysis using a threshold of r > 0.8. Although LPL and LPD describe the petiole, their high correlation with LA indicated redundant information. For the remaining 13 traits (both quantitative and binary qualitative variables), the Gower distance was used to accommodate mixed data types, and agglomerative hierarchical clustering was performed with the average linkage method, as shown in Figure 1.

The dendrogram revealed clear phenotypic differentiation among the accessions. At a dissimilarity threshold of 25, the 24 samples were divided into two clusters, with Xuanwei (S15) separating as a single-sample cluster. At a threshold of 20, four clusters were obtained, among which S9, S15, and S17 each formed a separate single-sample cluster. These thresholds illustrate the hierarchical structure and highlight accessions diverging at high dissimilarity levels. The accessions were grouped into five clusters at a threshold of 15. This level marked a significant increase in fusion distance and resulted in the most biologically interpretable grouping of the accessions. The first cluster (17 accessions) was characterized by purplish-red flowers and small fruit length-to-width ratios. The second cluster (S1, S6, S12, and S14) featured a small leaf area, round and smooth leaves, fewer marginal teeth, and purplish-red stem nodes. The third cluster (S17 only) was distinguished by many marginal teeth, long stem nodes, and a high fruit length-to-width ratio. The fourth cluster (S9 only) had milky-white flowers, and the fifth cluster (S15 only) was characterized by short stem nodes.

### 2.4. Characterization of Transcriptome-Derived SSR Loci in C. asiatica

**Figure 2:**
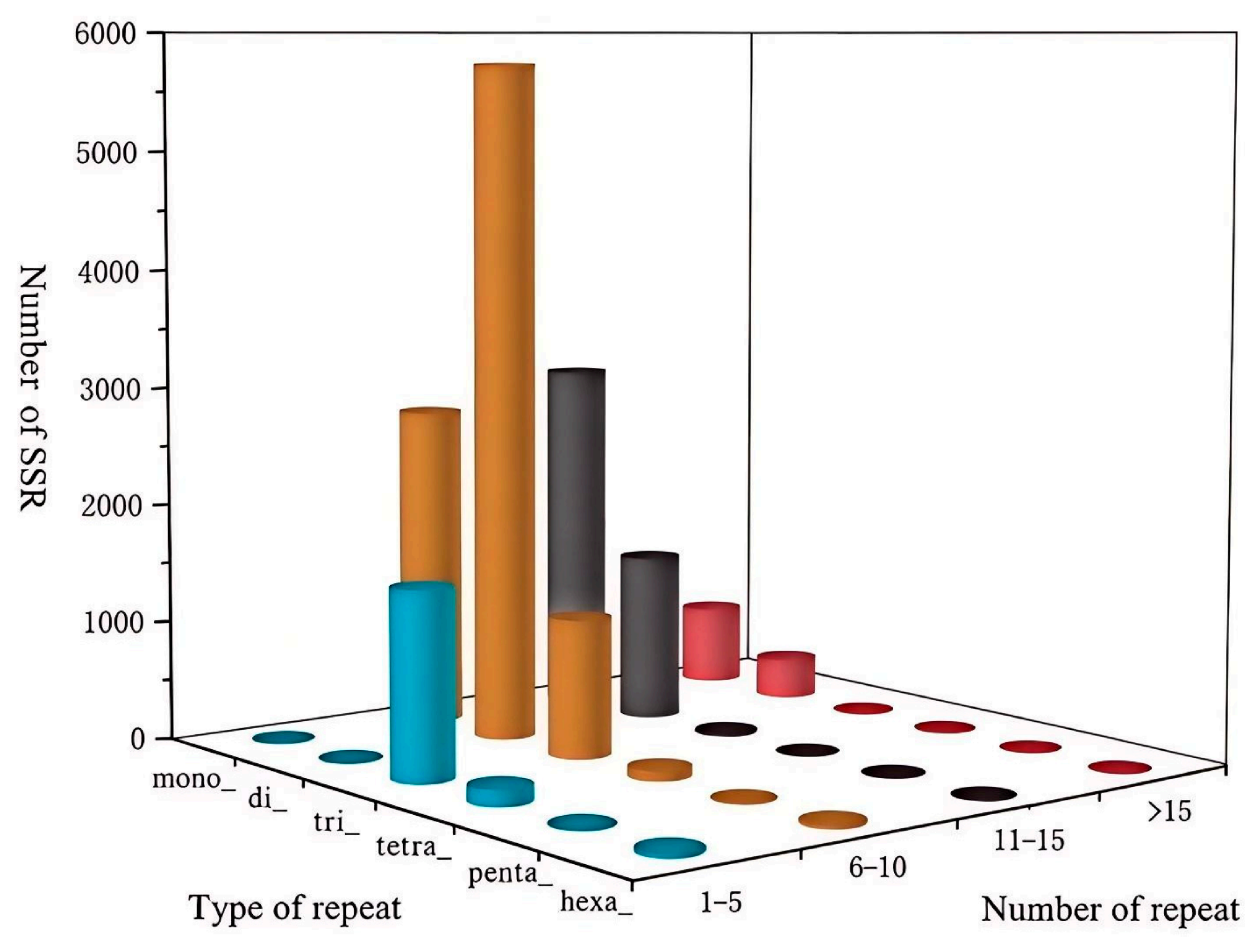
Distribution of SSR loci according to motif type and repeat number in the *C. asiatica* transcriptome.

To develop molecular markers suitable for genetic diversity analysis, we performed de novo transcriptome assembly and mined for SSR loci. The clean reads obtained from *C. asiatica* sequencing were *de novo* assembled and optimized using Trinity, resulting in 64,496 unigenes. A total of 16,799 SSR loci were identified using MISA, with a frequency of 26.05% in the *C. asiatica* transcriptome. Among the SSR repeat motifs, dinucleotide repeats were the most abundant and represented the dominant motif type, accounting for 44.31% of all SSR loci, with a frequency of 11.54%. Mononucleotide repeats were the second most common type, accounting for 37.64%, whereas trinucleotide repeats accounted for 16.19%. Tetranucleotide to hexanucleotide repeat motifs were detected at much lower frequencies, with pentanucleotide repeats being the least frequent, accounting for only 0.04%. As shown in Figure 2, the distribution of SSRs in the *C. asiatica* transcriptome varied among motif types and repeat numbers. Dinucleotide repeats with 6–10 repeat units were the most frequent category, accounting for 34.16% of all SSRs. Mononucleotide repeats with 11–15 and 6–10 repeat units followed, accounting for 17.72% and 16.05%, respectively. For trinucleotide-to-hexanucleotide repeats, loci with 1–5 repeat units predominated, and their length decreased as the repeat number increased. Taken together, dinucleotide repeat motifs were more abundant than the other five SSR motif types in *C. asiatica*, both in total number and repeat frequency. This indicates that dinucleotide repeats are potential makers *C. asiatica* and related species.

### 2.5. Analysis of SSR Primer Polymorphism in C. asiatica

**Figure 3:**
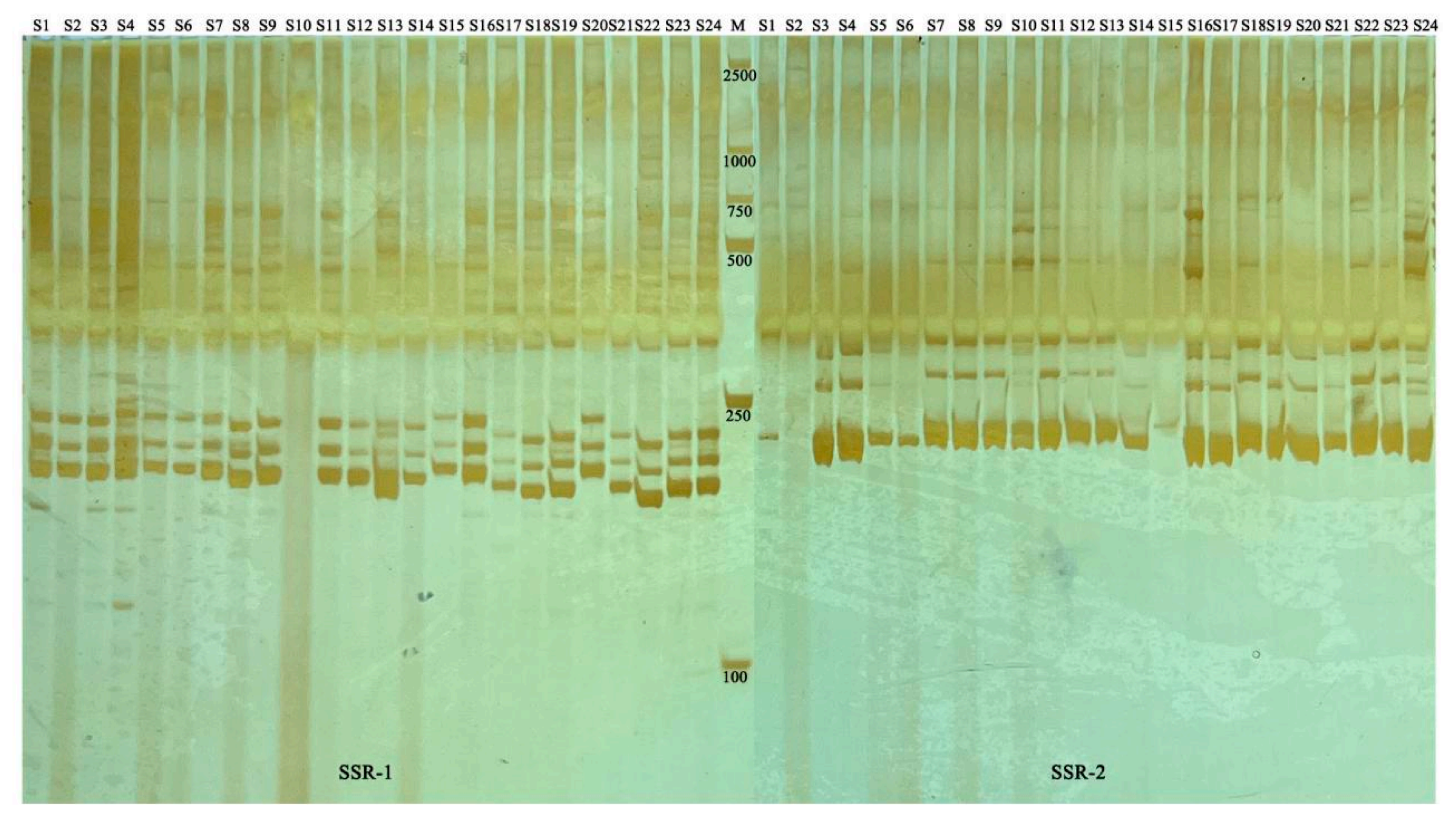
Representative polyacrylamide gel electrophoresis profiles of SSR-1 and SSR-2 in the 24 *C. asiatica* accessions. Lanes S1–S24 correspond to the 24 accessions, and lane M represents the DNA size marker. Clear and reproducible major bands were scored as alleles, whereas faint stutter bands and non-specific products were excluded from the analysis.

Polymorphism analysis was carried out on the 14 SSR loci retained after rescreening, using 24 C. asiatica accessions from different regions. The 14 polymorphic SSR loci used for genotyping are listed in Table A3. Figure 3 shows the amplification patterns of the primers SSR-1 and SSR-2 representative examples of the 14 polymorphic SSR markers. These two SSR markers were selected because SSR-1 displayed the highest number of alleles (Na = 6) and SSR-2 exhibited a typical stutter band pattern, together illustrating the range of allelic variation and the scoring methodology applied. Based on the amplification results, the genetic diversity indices for the 24 accessions are summarized in Table A10. The amplified products were separated by denaturing polyacrylamide gel electrophoresis and visualized by silver staining. As shown in Figure 3, multiple bands were observed in each lane.

Based on this scoring, PCR amplification of the 24 samples showed that 14 polymorphic SSR loci were present. A total of 52 alleles (Na) were detected across the 14 loci. The number of alleles per locus ranged from 3 to 6, with an average of 3.71. Among these loci, SSR-1 had the highest number of alleles (6). The number of effective alleles (Ne) ranged from 2.25 to 5.67, with an average of 3.15, and the mCaCIR028 locus had the lowest number of effective alleles. Observed heterozygosity (Ho) ranged from 0.00 to 0.83, with a mean of 0.25, while expected heterozygosity (He) ranged from 0.17 to 1.00, with a mean of 0.76. *Nei’s* genetic diversity index (H) varied from 0.55 to 0.82, with an average of 0.67, and Shannon’s information index (I) ranged from 0.94 to 1.76, with an average of 1.19. The polymorphism information content (PIC) of the primers ranged from 0.49 (mCaCIR028) to 0.80 (SSR-1), as shown in Table A10, with an average of 0.61.

### 2.6. Analysis of Genetic Similarity and Genetic Distance of C. asiatica SSR Markers

The genetic similarity and genetic distance among 24 *C. asiatica* germplasm resources from Yunnan Province were analyzed, and the results are shown in Table A11. The genetic similarity coefficients of *C. asiatica* ranged from 0.38 to 0.95, while the genetic distances ranged from approximately 0.05 to 1.02. Among them, the samples from Mengzi (S23) and Kaiyuan (S24) had a genetic similarity coefficient of 0.92 and a genetic distance of 0.08, indicating that they were the most closely related pair among all samples. In contrast, the samples from Eryuan (S2) and Kaiyuan (S24) had a genetic similarity coefficient of 0.38 and a genetic distance of 1.02, suggesting substantial genetic divergence. These results suggest that the *C. asiatica* germplasm collected from Yunnan comprises both closely related and highly differentiated genetic backgrounds.

### 2.7. Cluster Analysis of C. asiatica SSR Markers

**Figure 4:**
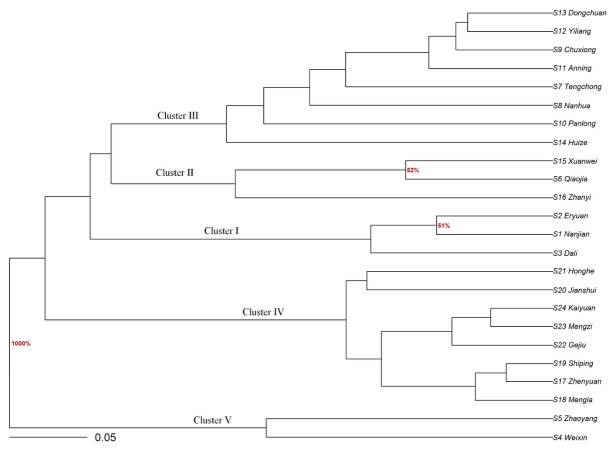
UPGMA clustering tree of 24 *C. asiatica* accessions based on *Nei’s* genetic distances. Accession labels include sampling site names. Numbers at nodes are bootstrap support values (%) from 1000 replicates (only values ≥50% are shown).

To clarify the genetic relationships among the accessions, a UPGMA dendrogram was constructed from *Nei’s* genetic distances (Table A11; Figure 4). Bootstrap support values (1000 replicates) are indicated at the nodes; only values ≥ 50% are displayed. At a genetic distance of 0.71, the 24 accessions were separated into two major groups. Weixin (S4) and Zhaoyang (S5) formed one group, while the remaining 22 accessions formed the other. At a genetic distance of approximately 0.55, five clusters were resolved. Cluster I comprised Eryuan (S2), Nanjian (S1), and Dali (S3), all from Plant Region I; S2 and S1 grouped with 51% bootstrap support. Cluster II contained Qiaojia (S6), Xuanwei (S15), and Zhanyi (S16); the pairing of S15 and S16 was supported by 52%. Cluster III included Dongchuan (S13), Yiliang (S12), Chuxiong (S9), Anning (S11), Tengchong (S7), Nanhua (S8), Panlong (S10), and Huize (S14). Cluster IV was the largest group, comprising Honghe (S21), Jianshui (S20), Kaiyuan (S24), Mengzi (S23), Gejiu (S22), Shiping (S19), Zhenyuan (S17), and Mengla (S18); within this cluster, Mengzi (S23) and Kaiyuan (S24) showed the smallest genetic distance (0.08). Cluster V consisted exclusively of Weixin (S4) and Zhaoyang (S5). Overall, the clustering showed partial correspondence with the floristic regions of Yunnan. However, because most internal nodes received low bootstrap support (<50%), the inferred groupings should be regarded as tentative and validated with additional markers.

### 2.8. Analysis of the Chemical Composition Diversity of C. asiatica

#### 2.8.1. Establishment and Analysis of *C. asiatica* Fingerprint Profiles

**Figure 5:**
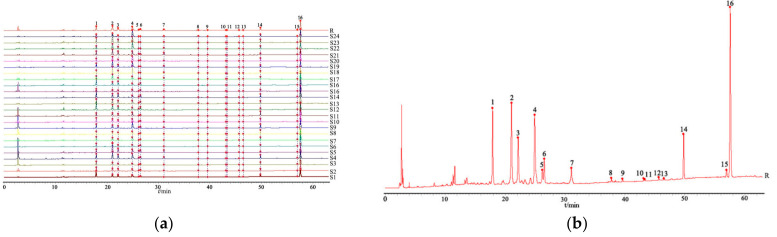
Fingerprint overlay (**a**) and control fingerprint (**b**) of *C. asiatica* resources from 24 different regions in Yunnan Province. The overlay displays superimposed chromatograms of all samples, with different colors representing individual regions. The control fingerprint (R) was generated using the median method, with Eryuan (S2) as the reference chromatogram. The *X*-axis indicates retention time, and the *Y*-axis indicates absorbance response. Dashed lines connect the 16 common peaks calibrated across samples. Among them, 5 peaks were identified by comparison with reference standards: peak 6 (madecassoside), peak 7 (asiaticoside), peak 9 (kaempferol), peak 11 (madecassic acid), and peak 12 (asiatic acid).

To evaluate the chemical consistency and variation among accessions, HPLC fingerprinting was performed. Common peaks were identified in the raw HPLC data from 24 *C. asiatica* samples collected across different regions of Yunnan Province. Reference fingerprint profiles (R) and superimposed HPLC fingerprints (S1–S24) were generated for all samples, and a total of 16 common peaks were marked. Matched peaks were connected using dashed lines. By comparing the DAD spectra and retention times with reference standards, five chromatographic peaks were identified: madecassoside (Peak 6), asiaticoside (Peak 7), kaempferol (Peak 9), madecassic acid (Peak 11), and asiatic acid (Peak 12). Figure 5 shows the overlay of HPLC fingerprints for the 24 *C. asiatica* samples together with the reference profile (R). Using this common pattern (R) as the reference spectrum, similarity values between individual sample fingerprints and R ranged from 0.884 to 0.988. The sample from Yiliang (S12) was an exception, with a similarity below 0.9. The remaining samples all had similarity values above 0.9, indicating generally high consistency among *C. asiatica* samples from Yunnan Province, except for Yiliang (Table A12).

**Figure 6:**
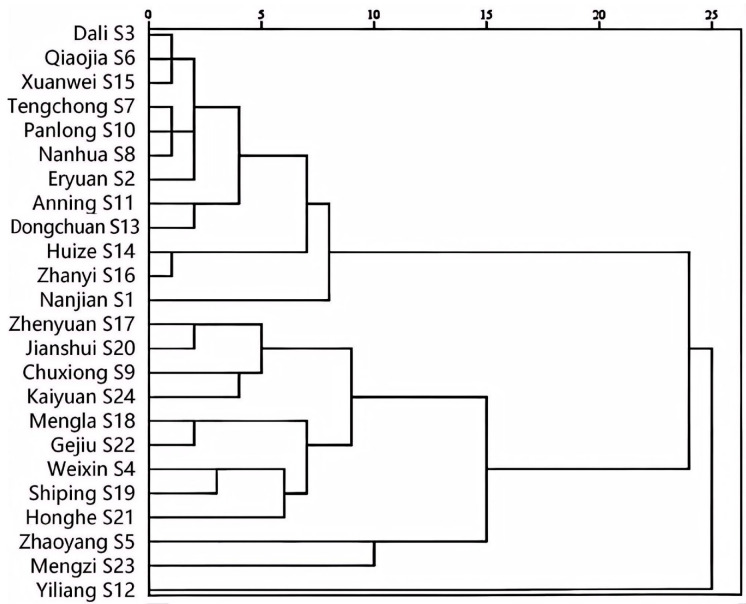
Cluster analysis of HPLC fingerprint profiles of *C. asiatica* from 24 different regions in Yunnan Province.

Hierarchical clustering was performed on 16 common peaks using the average linkage method and cosine distance as the dissimilarity measure in SPSS Statistics, version 20.0. When the inter-cluster distance was set to 25, the 24 samples grouped into two main clusters. Notably, the Yiliang sample (S12) formed a separate cluster, while the remaining 23 samples clustered together, indicating that its chemical composition differs considerably from that of the other regions. This observation is consistent with the similarity analysis, which showed that the Yiliang (S12) sample had the lowest similarity to the reference fingerprint (0.884; Table A12). Reducing the inter-cluster distance to 20 divided the samples into three clusters, and further reducing it to approximately 15 resulted in four clusters. Specifically, Cluster 1 mostly consisted of samples from Nanjian, Eryuan, Dali, Qiaojia, Tengchong, Nanhua, Panlong, Anning, Dongchuan, Huize, Xuanwei, and Zhanyi. Cluster 2 included samples from Weixin, Chuxiong, Zhenyuan, Mengla, Shiping, Jianshui, Gejiu, and Honghe, and Kaiyuan. Cluster 3 contained only two samples, Zhaoyang (S5) and Mengzi (S23), while Cluster 4 was the Yiliang (S12) sample. This clustering pattern highlights the distinct chemical profiles of samples from different regions, with the Yiliang sample standing out as particularly unique. The hierarchical clustering dendrogram is shown in Figure 6.

#### 2.8.2. Chemical Analysis of *C. asiatica*

High-performance liquid chromatography (HPLC) was used to analyze the samples according to the methods for preparing test solutions, chromatographic conditions, and elution programs. All the chromatograms were recorded, and the contents of five active components—asiaticoside, madecassoside, asiatic acid, madecassic acid, and kaempferol were determined by the external standard method (Table A13). The quantities of these substances varied considerably among the 24 sampling sites. Asiaticoside had the highest content, with concentrations ranging from 3.074 to 18.846 mg·g^−1^, followed by madecassoside (0.324–1.412 mg·g^−1^). Kaempferol had the lowest content, with concentrations ranging from 0.038 to 0.107 mg·g^−1^. The samples from Zhaoyang (S5) had the largest total amount of the five compounds, with a content of 20.175 mg·g^−1^. High total contents were also found in samples from Honghe (S21), Weixin (S4), Shiping (S19), Zhenyuan (S17), and Gejiu (S22), all exceeding 10 mg·g^−1^ (16.786, 15.059, 13.215, 11.603, and 10.318 mg·g^−1^, respectively). In contrast, the total contents of Qiaojia (S6), Nanjian (S1), Dali (S3), and Tengchong (S7) samples were less than 5 mg·g^−1^. The other areas have total contents varying from 5.279 to 9.703 mg·g^−1^. Asiaticoside content was highest in Zhaoyang (S5) at 18.846 mg·g^−1^, followed by Honghe (S21), Weixin (S4), and Shiping (S19) at 15.563, 13.93, and 12.284 mg·g^−1^, respectively. The content of madecassoside was greatest in Huize (S14) with 1.412 mg·g^−1^, while Tengchong (S7) had the smallest content (0.324 mg·g^−1^). Asiatic acid was maximum in Zhenyuan (S17) with 0.922 mg·g^−1^, and minimum in Qiaojia (S6) and Honghe (S21) with 0.083 and 0.072 mg·g^−1^, respectively. madecassic acid was the lowest among the four triterpenoid compounds, with all values below 0.5 mg·g^−1^. Jianshui (S20) had the highest level at 0.408 mg·g^−1^. Kaempferol was generally low, with the highest concentrations detected in Honghe (S21) and Huize (S14) at 0.107 and 0.103 mg·g^−1^, respectively, and below 0.1 mg·g^−1^ in the other regions. Overall, HPLC analysis revealed substantial interregional differences in the contents of triterpenoid and flavonoid components in *C. asiatica*. Asiaticoside was the predominant compound, while kaempferol was the least abundant. The sample from Zhaoyang (S5) had the highest total content, whereas samples from Qiaojia (S6) and three other regions had total contents below 5 mg·g^−1^. High-content samples for different compounds were distributed across different regions, suggesting pronounced regional dependence in the accumulation of these bioactive constituents.

## 3. Discussion

In this study, we integrated phenotypic characterization, SSR molecular markers, and HPLC-based chemical profiling to provide a comprehensive evaluation of the diversity present in 24 *C. asiatica* accessions collected across eight floristic regions of Yunnan Province, China. Our results revealed substantial phenotypic variation, moderate to high genetic diversity with partial geographic correspondence, and significant inter-accession differences in the accumulation of five key bioactive triterpenoids and flavonoids. Notably, the clustering patterns derived from morphological, genetic, and chemical datasets were not fully congruent, suggesting that the diversity architecture of *C. asiatica* is shaped by a complex interplay of genetic differentiation, environmental adaptation, and possibly independent selection pressures acting on distinct trait categories. Below, we discuss the implications of these findings for germplasm conservation, selective breeding, and the ecological adaptation of this medicinally important species.

Phenotypic characterization remains an important first step in germplasm evaluation because morphological traits reflect the combined effects of genetic background, developmental processes, and environmental conditions. In this study, 17 morphological characteristics were evaluated in 24 accessions of *C. asiatica* to investigate phenotypic diversity. The coefficient of variation (CV) for the quantitative characters varies from 8.8% to 48.4%, indicating different levels of phenotypic variability among accessions. The petiole length has the highest CV and shows substantial variation among the accessions. For the five qualitative characters, the Shannon-Wiener index (H’) ranged from 2.20 to 3.14, indicating varying levels of diversity among traits rather than uniformly high diversity. In particular, the index was influenced by the presence of rare categories within highly skewed distributions, as observed for flower color. Generally, *C. asiatica* in Yunnan Province exhibits a wide range of phenotypic variation, with reniform leaves and purplish-red flowers as the dominant characteristics. The correlation analysis among the 17 traits indicates significant or highly significant positive correlations between some leaf morphological traits. Additionally, correlations involving binary qualitative traits should be interpreted with caution, as their strength and direction may be influenced by the numerical coding (0/1) applied. PCA further confirmed that leaf morphology was the major source of phenotypic variation among the accessions; the first five components accounted for 82.54% of the total variance and were mainly associated with leaf size and vegetative development. These findings are consistent with previous studies emphasizing the importance of leaf morphological traits for comprehensive germplasm characterization [14,20]. It should be noted that some qualitative traits showed uneven distribution across phenotypic categories, which may be partly attributable to the limited sample size and the species’ inherent growth characteristics.

The phenotypic variation observed in Yunnan *C. asiatica* accessions is consistent with previous reports of morphological differentiation in this species. For instance, Alqahtani et al. [14] demonstrated that leaf morphology and stem growth habit are key traits distinguishing *C. asiatica* from its closely related species in Australia, and suggested that morphological differences among accessions from different geographic origins may reflect genetic adaptation to local environments. Similarly, in the present study, accessions from different floristic regions exhibited distinct phenotypic characteristics, particularly in leaf size, leaf shape, and stem node color, supporting the notion that phenotypic traits are shaped by both genetic differentiation and environmental adaptation.

The 24 accessions employed in our research were obtained from eight phytogeographic regions in Yunnan, which included the natural distribution of *C. asiatica* in the province and thus ensured representativeness. Phenotypic trait clustering separated the 24 accessions into five groups, whereas clustering based on SSR molecular markers was closer to the origin regions. Comparison of the phenotypic and SSR-based clustering revealed only partial overlap, suggesting that morphological variation is shaped by both genetic and environmental factors. The first phenotypic cluster comprised most of the samples; except for Zhenyuan, all the accessions in the fourth and fifth SSR clusters were also included in this cluster. The other SSR clusters had little association with the phenotypic clusters. The lack of full concordance may reflect the limited number of SSR loci examined and the polygenic nature of the phenotypic traits. The selected SSR loci may not adequately capture the genetic basis of the phenotypic traits, or the traits may not be clearly expressed under common-garden conditions. Moreover, environmental factors exert a significant influence on phenotypic expression, leading to discrepancies between the two clustering methods. Both phenotypic traits and SSR markers can be applied for genetic relationship analysis of germplasm, but the former is more influenced by environmental conditions and has some limitations compared to DNA-based markers in measuring genetic diversity. Future studies employing a Mantel test to statistically correlate the genetic and phenotypic distance matrices would help clarify the relationship between genetic similarity and morphological resemblance.

The expected heterozygosity (He = 0.76) and Shannon’s information index (I = 1.19) observed in this study were higher than those reported in previous SSR-based studies of *C. asiatica*, while the average number of alleles per locus (Na = 3.71) was within the range of previously reported values. Mathavaraj and Sabu [21] reported lower genetic diversity (H = 0.46, I = 0.83) among 80 accessions from southern India using 10 genomic SSR markers, and Rakotondralambo et al. [17] reported a higher Na (4.3) but considerably lower He (0.379) among Madagascan populations. These differences may reflect variations in geographic scale, population structure, or marker type. The present study also detected higher polymorphism than that reported by Sakthipriya et al. [18] and Rohini et al. [19], who found moderate to low diversity in Indian *C. asiatica* germplasm. The relatively high genetic diversity observed in Yunnan accessions may be attributable to the region’s complex topography, diverse climatic conditions, and long history of natural adaptation. Additionally, the partial correspondence between SSR-based clusters and floristic regions observed here is consistent with the findings of Mathavaraj and Sabu [21], who reported significant population differentiation and a genetic structure influenced by geographic distribution in Indian *C. asiatica* populations. However, it should be noted that the majority of internal nodes in our UPGMA dendrogram received low bootstrap support (<50%), indicating that the inferred groupings, particularly the deeper branches, should be regarded with caution. Several factors may contribute to this topological uncertainty. First, the limited number of SSR loci (*n* = 14) may provide insufficient resolution to fully resolve fine-scale genetic relationships, especially among closely related accessions. Second, the relatively small sample size per geographic region may reduce statistical power for detecting robust population subdivisions. Third, *C. asiatica* is a widespread, creeping herb with potential for both clonal propagation and long-distance seed dispersal, which could result in ongoing gene flow among populations and blur the genetic boundaries between geographic groups. The observed partial correspondence, therefore, likely reflects an interplay between historical differentiation shaped by geographic isolation and contemporary gene flow homogenizing local genetic structure. Future studies employing genome-wide SNP markers and larger population samples would be valuable to validate and refine the genetic structure inferred here, and to distinguish the relative contributions of gene flow versus genetic drift in shaping the diversity patterns observed in Yunnan *C. asiatica*.

The chemical variation observed among Yunnan accessions aligns with previous reports on the influence of geographic origin, plant organ, and harvest period on triterpenoid accumulation. Puttarak and Panichayupakaranant [15] demonstrated that pentacyclic triterpene content in *C. asiatica* varies significantly according to geographic origin and harvest season, with leaves containing the highest concentrations. Similarly, Alqahtani et al. [14] found that chemical composition, together with morphological and molecular markers, effectively differentiated Centella species and accessions. In the present study, the substantial variation in asiaticoside content (3.07–18.85 mg·g^−1^) across Yunnan accessions confirms the strong regional dependence of bioactive compound accumulation. Deng et al. [20] recently developed EST-SSR markers and observed genetic differentiation in terpenoid biosynthesis-related genes among *C. asiatica* populations, suggesting that the chemical variation documented here may have an underlying genetic basis that warrants further investigation. Future studies integrating transcriptomic and metabolomic approaches, as demonstrated by Wan et al. [22], could help elucidate the molecular mechanisms driving differential triterpenoid accumulation in Yunnan *C. asiatica* germplasm.

*C. asiatica* is a traditional Chinese medicine that mainly consists of triterpenoid and flavonoid components. Using HPLC, the chemical fingerprint patterns of 24 samples from Yunnan Province were obtained, and 16 common peaks were detected. Among them, five important substances—asiaticoside, madecassoside, asiatic acid, madecassic acid, and kaempferol—were identified. The results showed that the amounts of these substances varied among different samples. Asiaticoside was the predominant compound, whereas kaempferol was the least abundant. Some differences from previous research may be attributed to variations in sample sources, collection times, analytical instruments, and handling methods. Additionally, regional differences were observed: samples from regions with lower temperatures and higher humidity tended to show higher total concentrations of the five substances. Although this observation requires validation through quantitative environmental correlation analysis, it suggests that the environmental conditions have a great effect on the chemical composition of *C. asiatica*. Further studies concentrating on specific environmental factors may help to explain the reasons for these regional variations.

It is worth noting that while high-throughput SNP genotyping methods have become increasingly accessible, SSR markers remain a valuable and widely used tool for germplasm evaluation, particularly for species with limited genomic resources at the time of study initiation. In the case of *C. asiatica*, although a reference genome and transcriptomic datasets are now publicly available, the SSR markers employed here offer several practical advantages: (i) low cost and technical simplicity, making them suitable for breeding programs in developing regions; (ii) high allelic diversity per locus, which is beneficial for discriminating closely related accessions; and (iii) direct comparability with previously published *C. asiatica* genetic diversity studies that also relied on SSR markers [18,19,20,21]. Future studies incorporating larger population samples and genome-wide markers, such as SNPs or high-density EST-SSR panels, would provide greater resolution for assessing the population structure of *C. asiatica*. Such approaches would also allow a more rigorous evaluation of the relative contributions of genetic drift, gene flow, and environmental heterogeneity to the distribution of genetic diversity within Yunnan germplasm.

## 4. Materials and Methods

### 4.1. Collection of C. asiatica Germplasm Resources

**Figure 7:**
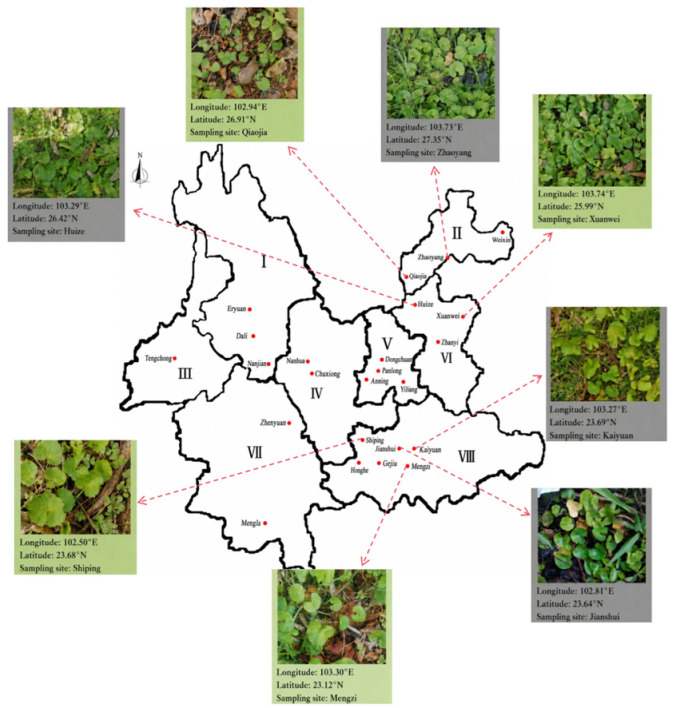
Geographic distribution of the 24 *C. asiatica* collection sites in Yunnan Province, China. Collection sites are plotted on the map and color-coded according to the eight floristic regions (I–VIII) defined by Li et al. (2015) [35]. In situ photographs of eight representative accessions are connected by arrows to their corresponding locations. The photographs illustrate the plants’ vegetative state at the time of collection and are not intended for morphological comparison. Detailed collection information for all accessions is provided in Table A1.

We gathered the germplasm of *C. asiatica* from various sites in Yunnan Province. First, the distribution records of wild *C. asiatica* were obtained from the Chinese Virtual Herbarium (CVH, www.cvh.ac.cn (accessed on 15 June 2026)), a database managed by the Institute of Botany, Chinese Academy of Sciences. These records were verified and further supplemented with the collection sites mentioned in the literature. The sampling locations were selected according to the vegetation zoning system for Yunnan proposed by Li et al. (2015) [35], which combines floristic composition with phylogenetic structure. To ensure proper geographical distribution, we ensured that each type of vegetation had at least one sampling point. The detailed collection information is shown in Figure 7.

Based on this zoning, 24 accessions were collected from sites selected to maximize geographic and ecological coverage across Yunnan Province. Selection criteria included: (i) representation of all eight floristic regions defined by Li et al. (2015) [35], (ii) an altitudinal range from 464 m to 2106 m, (iii) the inclusion of distinct climatic zones (subtropical to temperate), and (iv) the availability of wild populations with sufficient plant material for transplantation and analysis.

### 4.2. Morphological Characterization and Statistical Analysis of C. asiatica

Phenotypic characterization was conducted using a simple random sampling design. Five plants of each accession were randomly selected, and 17 morphological traits—12 quantitative and 5 qualitative—were measured based on key botanical descriptors of the species [36]. Different measurement instruments were employed depending on the trait: a measuring tape (Delixi Electric Co., Ltd., Wenzhou, China) for length-related traits (e.g., LPL, LL, LW, SL), and a Vernier caliper (Guilin Measuring & Cutting Tool Co., Ltd., Guilin, China) for traits requiring greater precision (e.g., LPD, SD, FL, FW). Leaf area (LA) was calculated from leaf length and width, and all data were acquired in adherence to the criteria summarized in Table A2.

Quantitative traits were processed by computing descriptive statistics and the Shannon–Wiener diversity index, while qualitative traits were assessed through frequency distributions and the Shannon–Wiener diversity index. A detailed set of analyses was conducted on all 17 phenotypic traits using IBM SPSS Statistics software, version 27.0 (IBM Corp., Armonk, NY, USA). Spearman’s rank correlation analysis was performed to assess relationships among all 17 traits (quantitative and qualitative), and the resulting coefficients are reported in Table A7. PCA was performed, and the variances and cumulative contributions of individual principal components were calculated. PCA scores were obtained for each population. To remove scale effects, the data were normalized prior to clustering; agglomerative hierarchical clustering was then performed using the Gower distance, a suitable dissimilarity measure for mixed data types.

### 4.3. RNA Extraction, Transcriptome Assembly, and SSR Identification

Total RNA was extracted using a plant RNA extraction kit (Sangon Biotech, Shanghai, China) according to the manufacturer’s protocol. RNA quality and integrity were verified by agarose gel electrophoresis and spectrophotometry. Transcriptome sequencing and de novo assembly were performed by a commercial service provider (Majorbio Bio-Pharm Technology Co., Ltd., Shanghai, China) using the Illumina sequencing platform and Trinity assembler, respectively, yielding 64,496 unigenes. A total of 16,799 SSR loci were identified from these unigenes using the MicroSAtellite (MISA) software (v1.0) with the following criteria: a minimum of 10 repeats for mononucleotide motifs, a minimum of 6 repeats for dinucleotide motifs, and a minimum of 5 repeats for trinucleotide to hexanucleotide motifs. The frequency, average distribution distance, motif types, and repeat numbers of SSRs were calculated and summarized using descriptive statistics.

### 4.4. SSR Primer Source, Primer Design, and Screening

Two sets of SSR primers were evaluated in this study. The first set consisted of ten previously published *C. asiatica* SSR loci [17], whose amplification performance had been firmly validated. However, preliminary testing revealed that several published primers showed low polymorphism or inconsistent amplification in the Yunnan accessions, providing insufficient marker density to fully capture the genetic diversity of the collection. Therefore, a second set of 20 newly designed loci was developed from the transcriptome data of this study, resulting in a total of 30 candidate SSR loci. Detailed PCR amplification conditions and primer sequences are shown in Table A3 and Table A4. An initial screening was performed using eight accessions representing distinct geographic regions. All 30 candidate SSR loci were evaluated for amplification efficiency, reproducibility, and level of polymorphism. After this screening, 15 loci produced clear, reproducible, and polymorphic amplification profiles and were therefore selected for genotyping. However, mCaCIR002 was subsequently excluded from the final analysis due to inconsistent amplification across samples. As a result, 14 loci remained for genotyping (Table A3).

### 4.5. Plant Material, DNA Extraction, and SSR-PCR Amplification

In November 2022, young, healthy, disease- and pest-free leaves were sampled from the transplants at the Southwest Forestry University experimental site (Panlong District, Kunming, Yunnan), representing all 24 accessions. Leaves were labeled, placed in cryovials, flash-frozen in liquid nitrogen, and stored at –80 °C. Genomic DNA was extracted with the TSINGKE TSP102-200 High-Efficiency Plant Genomic DNA Extraction Kit (TSINGKE Biotechnology Co., Ltd., Beijing, China). The extracted DNA was subsequently used as a template in PCR reactions to amplify the SSR loci listed in Table A3. All PCR amplifications were carried out following the thermal cycling program detailed in Table A4. The consistency of amplification conditions with those provided in the tables was verified during the experimental workflow.

### 4.6. Polymorphism Detection by Polyacrylamide Gel Electrophoresis

After separation of the PCR products on 10% non-denaturing polyacrylamide gels, they were observed by silver staining. After electrophoresis, each gel was fixed by shaking gently, washed in deionized water, stained with a silver nitrate (Sinopharm Chemical Reagent Co., Ltd., Shanghai, China) solution (protected from light) that had been freshly prepared, washed again briefly, and developed until the bands became visible enough. The stained gels were removed from the gel box for photographing on a light box to document support further study.

### 4.7. SSR Data Processing and Analysis for C. asiatica

The 14 selected SSR loci were used to genotype all 24 accessions. In addition to one or two major allelic bands (target amplicons), a series of fainter stutter bands usually appeared, mainly resulting from slipped-strand mispairing of the DNA polymerase during PCR amplification. Occasionally, non-specific amplification products were also visible. To ensure reliable genotyping, a strict scoring standard was applied: only clear, reproducible bands corresponding to the expected SSR amplicons were scored as alleles; stutter bands and faint non-specific products were disregarded. Homozygous individuals were scored as a single allele (one band), and heterozygous individuals were scored as two distinct alleles (two bands of equal or similar intensity). Allele sizes were determined by comparison with a known DNA size marker. Allele counts (Na), effective allele numbers (Ne), observed heterozygosity (Ho), expected heterozygosity (He), *Nei’s* gene diversity index (H), and Shannon’s information index (I) were all derived using POPGENE version 1.32 (University of Alberta, Edmonton, Canada). Polymorphism information content (PIC) was estimated with PowerMarker V3.25 (North Carolina State University, Raleigh, NC, USA). Pairwise genetic similarity coefficients and *Nei’s* genetic distances among the 24 accessions were calculated based on SSR genotypes. A UPGMA dendrogram was constructed using NTSYS-pc version 2.1 (Applied Biostatistics Inc., Setauket, NY, USA). Bootstrap support values for the dendrogram were calculated using the ape package [37] in R with 1000 replicates.

### 4.8. Quantification of Major Bioactive Constituents of C. asiatica by HPLC

#### 4.8.1. Materials and Instrumentation

The analyses were conducted on 24 accessions from Yunnan Province using a high-performance liquid chromatography system (Thermo Fisher UltiMate 3000, Thermo Fisher Scientific, Munich, Germany). Other instruments employed were an ATX224 electronic analytical balance (Shimadzu, Kyoto, Japan), an ultrasonic cleaner (Kunshan Ultrasonic Instrument Co., Ltd., Kunshan, China), and a portable high-speed grinder (Wenling Lindai Machinery Co., Ltd., Wenling, China). The main reagents included chromatographic-grade acetonitrile and methanol (Shanghai Jizhi Biochemical Technology Co., Ltd., Shanghai, China), phosphoric acid (Guangdong Guanghua Technology Co., Ltd., Guangzhou, China), and reference standards (purity ≥ 98%) for asiaticoside (lot J30GB153193), madecassoside (lot D18J12S138013), asiatic acid (lot S04GB159773), madecassic acid (lot J12HB184580), and kaempferol (lot A01HB190000), all bought from Shanghai Yuanye Biotechnology Co., Ltd., Shanghai, China.

#### 4.8.2. HPLC Analysis and Fingerprinting

Peaks shared among the 24 chromatograms were identified using the Chromatographic Fingerprint Similarity Evaluation System for Traditional Chinese Medicine (Version 2012.130723); this identification step did not omit any common features. A reference fingerprint was generated using the median method, with the Eryuan (S2) accession as the reference chromatogram and a time-window width of 0.1 min—no wider tolerance was adopted. Following multi-point calibration and peak matching, superimposed HPLC fingerprints were constructed, not without ensuring alignment fidelity. For every accession, 1 g of sample material was extracted and subjected to analysis, and the concentrations of asiaticoside, madecassoside, asiatic acid, madecassic acid, and kaempferol were calculated by the external standard method—no alternative quantification strategy was employed.

## 5. Conclusions

This study provides a comprehensive evaluation of the phenotypic, genetic, and chemical diversity of 24 *C. asiatica* accessions collected across eight floristic regions in Yunnan Province, China. Substantial variation was observed at all three levels, with leaf morphology identified as the primary determinant of phenotypic diversity, moderate to high genetic diversity partially correlated with geographic origin, and significant inter-accession differences in bioactive compound content (4.09–20.18 mg·g^−1^). Among the evaluated materials, Zhaoyang (S5), Honghe (S21), and Weixin (S4) emerged as particularly promising accessions due to their relatively high contents of bioactive compounds. The partial overlap observed among phenotypic, genetic, and chemical clustering patterns highlights the importance of multi-level evaluation in germplasm characterization. These findings provide a scientific basis for the conservation and sustainable utilization of *C. asiatica* germplasm in Yunnan.

## Data Availability

The transcriptome sequencing data were generated by a third-party service (Majorbio Bio-Pharm Technology Co., Ltd., Shanghai, China), and the original fastq files are no longer retrievable. All assembled unigene sequences, SSR locus information, and primer sequences derived from the transcriptome are available in Appendix A of this article. All other data generated or analyzed during this study are included in this published article.

## Appendix A

**Table A1:** Collection sites and geographic information for the 24 *C. asiatica* accessions (S1–S24) sampled in Yunnan Province.

NO.	Collection Area	Chinese Name	East Longitude	North Latitude	Altitude (m)
S1	Nanjian	南涧	100°55′	24°81′	1733.71
S2	Eryuan	洱源	99°96′	26°12′	2046.58
S3	Dali	大理	100°13′	25°85′	1954.87
S4	Weixin	威信	104°91′	27°97′	892.40
S5	Zhaoyang	昭阳	103°73′	27°35′	1914.38
S6	Qiaojia	巧家	102°94′	26°91′	815.36
S7	Tengchong	腾冲	98°55′	25°11′	1688.96
S8	Nanhua	南华	101°27′	25°53′	1812.21
S9	Chuxiong	楚雄	101°23′	24°99′	2034.34
S10	Panlong	盘龙	102°77′	26°07′	1952.15
S11	Anning	安宁	102°38′	24°93′	1872.93
S12	Yiliang	宜良	103°13′	24°94′	1789.62
S13	Dongchuan	东川	103°17′	26°06′	1186.92
S14	Huize	会泽	103°30′	26°42′	2086.47
S15	Xuanwei	宣威	103°74′	25°99′	2105.93
S16	Zhanyi	沾益	103°80′	25°58′	1849.3
S17	Zhenyuan	镇沅	101°08′	24°05′	1042.39
S18	Mengla	勐腊	101°26′	21°92′	570.98
S19	Shiping	石屏	102°51′	23°68′	1383. 12
S20	Jianshui	建水	102°82′	23°16′	1824.67
S21	Honghe	红河	102°42′	23°34′	464.43
S22	Gejiu	个旧	103°16′	23°37′	1658.86
S23	Mengzi	蒙自	103°30′	23°12′	980.21
S24	Kaiyuan	开远	103°27′	23°70′	1100.75

**Table A2:** Quantitative and qualitative morphological traits recorded for *C. asiatica*, and their measurement standards.

Trait	English Abbreviation	Descriptors (Units of Measurement)
Leaf area	LA	Leaf blade area (cm^2^)
Petiole length	LPL	Distance from petiole insertion point to leaf base (cm)
Petiole diameter	LPD	Petiole cross-sectional length (mm)
leaf length	LL	Distance from leaf apex to leaf base (cm)
leaf width	LW	Maximum leaf blade width (cm)
Leaf length-to-width ratio	LL/LW	Leaf length/width ratio
Leaf marginal serration number	NLS	Number of leaf marginal serrations
Internode length	SL	Length of the connecting stem between two plants (cm)
Internode diameter	SD	Internode cross-sectional length (mm)
Fruit length	FL	Distance from fruit base to apex (mm)
Fruit width	FW	Maximum fruit width (mm)
Fruit length-to-width ratio	FL/FW	Fruit length/width ratio
Leaf shape	LS	orbicular (0), reniform (1)
Serration conspicuousness (of leaf margin)	DOLS	Leaf marginal serration conspicuousness: conspicuous (0), inconspicuous (1)
Leaf blade rugosity	WLSW	Leaf blade surface: smooth (0), rugose (1)
Stem node color	SC	Pale purple (0), pale purplish-red (1)
Flower color	FC	Purplish-red (0), milky white (1)

**Table A3:** List of SSR loci used for diversity analysis of *C. asiatica*.

SSR Locus	Repeat Motifs	Forward Primer (5′-3′)	Reverse Primer (5′-3′)	Product Size (bp)
mCaCIR002	(CT)_30_	CCACAGGTAACACCGAAT	GCACTTGCACTATCTGGAA	141–169
mCaCIR006	(CT)_15_	ACGGGCATTTATTCCATT	GCAAACCACCACAACTTC	215–219
mCaCIR007	(TG)_12_	GCGTGTCCTTTGGTGGTTATT	AGGGGATCAAACCTCATC	234–240
mCaCIR027	(CA)_6_(AG)_10_	ACCCCAAGACCTTCAGTT	CCTTCTGCTTTCCCTTTT	225–233
mCaCIR028	(AG)_5_(AG)_8_	CAGAGTTTGGGCAGAAAA	GACGAGTGGAGGATAAGAAA	197–203
mCaCIR029	(CA)_8_(AT)_5_(CT)_5_	GGTCTGAGGTCTGTTGAGG	CGCATTGACAGAACAAAA	317–366
mCaCIR030	(TG)_5_(TA)_5_(GA)_20_	GGCAAATCGAGAGCAATA	ACGGAAAAGCCTAACAGC	240–248
SSR-1	(TC)_18_	CGTGGGAGATGGAAAAGAAA	GAGAGGAGAGGTTGGGGAAC	197
SSR-2	(TCT)_12_	AATTGGAGACGACAACTGGG	AGACATAACCACCGCCAAAG	255
SSR-3	(TGTATG)_6_	ATGTGTGGCGTGAGTGTGAT	ACTTTCTCCAGGTACCGGCT	200
SSR-6	(CT)_23_	GCCATCTGCAGGTGTTATGA	AACCATGACCTTGGAAAGGA	265
SSR-7	(GA)_14_	CAGTGTCCAGCTCGAATGAA	CCAGGTCCTCCATTTTTCAG	254
SSR-8	(GA)_19_	AAAAAGACGGGGCGTAGAAT	CCTCCACCTACAACCTCCAA	164
SSR-10	(TTTA)_5_	AAGGTCAAGAAGCCCCAACT	TCTTGTTTCCAGGGCTTTTG	170
SSR-11	(TGTGC)_5_	CCGGATCTGCTGAGAAAAAG	TTGCCCAATCTCCAAGAAAC	211

Note: mCaCIR002 was excluded from the final analysis due to inconsistent amplification across samples; the remaining 14 loci were used for genotyping.

**Table A4:** SSR-PCR amplification program.

Procedure	Temperature	Duration	Cycles
Initial denaturation	95 °C	5 min	30
Denaturation	95 °C	30 s	30
Annealing	55–56 °C	30 s	30
Extension	72 °C	30 s	30
Extension	72 °C	8 min	
End	4 °C	10 min	

**Table A5:** Descriptive statistics and the Shannon–Wiener diversity index (H’) for the 12 quantitative traits of *C. asiatica*.

Trait	Abbreviation	Max	Min	Mean	S	CV	(H’)
Leaf area (cm^2^)	LA	11.91	2.10	6.58	3.07	46.70%	3.07
Petiole length (cm)	LPL	14.40	2.17	8.19	3.96	48.40%	3.06
Petiole diameter (mm)	LPD	1.80	0.83	1.34	0.24	18.10%	3.16
Leaf length (cm)	LL	3.17	1.33	2.26	0.55	24.30%	3.15
Leaf width (cm)	LW	5.33	2.10	3.65	0.98	26.90%	3.14
Length-to-width ratio	LL/LW	0.73	0.51	0.63	0.06	10.00%	3.17
Number of leaf edge serrations	NLS	36.00	18.00	26.00	4.39	17.10%	3.16
Internode length (cm)	SL	12.83	5.50	7.93	2.04	25.70%	2.64
Internode diameter (mm)	SD	1.52	0.83	1.22	0.17	14.00%	3.15
Fruit length (mm)	FL	3.27	2.27	2.87	0.25	11.40%	3.17
Fruit width (mm)	FW	3.77	2.06	3.24	0.37	8.80%	3.17
Fruit length-to-width ratio	FL/FW	1.91	0.80	0.94	0.23	24.10%	3.16
Average	-	-	-	-	-	22.96%	3.14

Note: S, standard deviation; CV, coefficient of variation; H’, Shannon–Wiener diversity index. Trait abbreviations are defined in Table A2.

**Table A6:** Classification and frequency distribution of 5 quality traits of *C. asiatica*.

Trait	H′	Trait Description	Number of Accessions	Frequency of Distribution (%)
Whether leaf margin serration is obvious	2.64	Obvious	10	41.67
		Not obvious	14	58.33
Leaf shape	3.04	Round	3	12.5
		Kidney-shaped	21	87.5
Whether the leaf surface is wrinkled	2.94	Smooth surface	5	20.83

**Table A7:** Correlation matrix of 17 phenotypic traits evaluated in 24 *C. asiatica* accessions from Yunnan Province. Spearman correlation coefficients are shown for all pairwise trait combinations.

Trait	LA	LPL	LPD	LL	LW	LL/LW	NLS	DOLS	LS	WLSW	SL	SD	**SC**	**FC**	**FL**	**FW**	**FL/FW**
LPL	0.855 **	1															
LPD	0.834 **	0.761 **	1														
LL	0.972 **	0.876 **	0.805 **	1													
LW	0.980 **	0.823 **	0.874 **	0.930 **	1												
LL/LW	−0.299	−0.115	−0.391	−0.108	−0.455 *	1											
NLS	0.634 **	0.606 **	0.629 **	0.621 **	0.698 **	−0.420 *	1										
DOLS	0.352	0.282	0.278	0.233	0.415 *	−0.560 **	0.377	1									
LS	0.408 *	0.260	0.477 *	0.387	0.454 *	−0.337	0.472 *	0.192	1								
WLSW	0.555 **	0.380	0.498	0.530 **	0.613 **	−0.418 *	0.625 **	0.191	0.737 **	1							
SL	0.281	0.477 *	0.200	0.362	0.248	0.150	0.625 **	0.287	0.108	0.112	1						
SD	0.475 *	0.445 *	0.647 **	0.416 *	0.568 **	−0.529 **	0.404	0.430 *	0.389	0.487 *	−0.003	1					
SC	−0.593 **	−0.595 **	−0.574 **	−0.621 **	−0.560 **	−0.062	−0.478 *	−0.044	−0.488 *	−0.450 *	−0.232	0.026	1				
FC	−0.003	−0.323	−0.049	−0.024	−0.031	0.043	−0.245	−0.176	−0.079	−0.107	−0.083	−0.152	0.162	1			
FL	0.658 **	0.559 **	0.627 **	0.638 **	0.709 **	−0.375	0.451 *	0.171	0.170	0.437 *	−0.010	0.398	−0.362	−0.295	1		
FW	0.392	0.071	0.329	0.329	0.415 *	−0.333	0.020	−0.031	0.194	0.359	−0.386	0.181	−0.023	0.134	0.677 **	1	
FL/FW	−0.150	−0.016	−0.098	−0.098	−0.164	0.292	0.022	0.141	0.058	0.110	0.026	0.168	−0.290	−0.124	−0.325	−0.491 *	1

Note: LA: Leaf area; LPL: Petiole length; LPD: Petiole diameter; LL: Leaf length; LW: Leaf width; LL/LW: Leaf length-to-width ratio; NLS: Number of leaf margin serrations; DOLS: Whether leaf margin serration is obvious; LS: Leaf shape; WLSW: Whether leaf surface is wrinkled; SL: Internode length; SD: Internode diameter; SC: Internode color; FC: Flower color; FL: Fruit length; FW: Fruit width; FL/FW: Fruit length-to-width ratio; **: Significant correlation at the 0.01 level; *: Significant correlation at the 0.05 level.

**Table A8:** Loadings of 17 phenotypic traits on the first five principal components (PC1–PC5) obtained from PCA of 24 accessions of *C. asiatica* from Yunnan Province.

Trait	Principal Component				
	PC1	PC2	PC3	PC4	PC5
Leaf width (LW)	0.970	−0.068	0. 111	−0.066	0.093
Leaf area (LA)	0.934	0.009	0.246	−0.064	0.113
Leaf length (LL)	0.898	0.134	0.357	−0.031	0.087
Petiole diameter (LPD)	0.893	−0.026	0.084	0.000	0.057
Petiole length (LPL)	0.830	0.324	0.232	−0.227	−0.153
Number of leaf margin serrations (NLS)	0.768	0.208	−0.223	−0.064	0.017
Fruit length (FL)	0.725	−0.370	0.250	−0.047	−0.409
Whether the leaf surface is wrinkled (WLSW)	0.708	−0.039	−0.271	0.444	0.082
Internode diameter (SD)	0.632	−0.099	−0.451	0.070	−0.082
Internode color (SC)	−0.628	−0.443	−0.191	−0.355	0.062
Leaf shape (LS)	0.572	0.049	−0.339	0.474	0.225
Fruit width (FW)	0.377	−0.780	0.296	0.223	−0.086
Fruit length-to-width ratio (FL/FW)	−0.109	0.650	−0.282	0.467	−0.125
Internode length (SL)	0.289	0.609	0.056	−0.473	0.270
Leaf length-to-width ratio (LL/LW)	−0.461	0.535	0.587	0.147	−0.070
Whether leaf margin serration is obvious (DOLS)	0.424	−0.052	−0.542	−0.547	0.114
Flower color (FC)	−0.175	−0.242	0.256	0.148	0.852
Characteristic value	7.504	2.266	1.675	1.454	1.131
Contribution rate (%)	44.14	13.33	9.86	8.56	6.66
Cumulative contribution rate (%)	44.14	57.47	67.33	75.88	82.54

**Table A9:** Rank of comprehensive score by 17 phenotypic traits of *C. asiatica*.

Region	F1	F2	F3	F4	F5	Total F	Rank
S11	0.744	−0.159	2.054	0.865	−0.224	0.570	1
S8	0.826	1.142	0.684	−0.922	0.851	0.562	2
S17	0.549	2.747	−1.195	−0.909	1.472	0.510	3
S22	1.189	−0.822	0.522	−0.194	0.075	0.455	4
S20	0.795	0.922	−0.241	−0.861	0.941	0.438	5
S24	1.188	−0.674	−0.109	0.115	0.009	0.434	6
S23	0.503	−0.334	1.110	0.596	0.042	0.341	7
S4	0.669	−0.339	−0.278	0.246	0.126	0.252	8
S21	0.962	−0.891	−0.691	0.054	−0.153	0.232	9
S3	−0.220	0.370	1.136	1.164	0.008	0.165	10
S19	0.706	−0.713	−0.515	0.236	−0.374	0.160	11
S9	0.794	1.140	−1.216	−0.697	−4.012	0.053	12
S10	0.312	−0.592	−0.033	−0.555	0.542	0.044	13
S15	−0.906	1.764	−0.979	3.010	−0.200	−0.016	14
S13	−0.680	0.928	0.932	0.119	0.100	−0.067	15
S7	−0.144	−0.496	0.055	0.410	0.187	−0.076	16
S2	−0.754	0.036	0.621	1.178	0.301	−0.145	17
S5	0.028	−0.897	−1.151	−0.273	0.371	−0.220	18
S12	−0.764	0.169	1.480	−1.159	−0.788	−0.321	19
S16	−0.657	−1.060	−0.724	0.728	0.127	−0.432	20
S18	−0.357	−1.451	−1.883	0.003	0.451	−0.506	21
S14	−1.504	−0.192	−0.924	−0.665	0.916	−0.776	22
S6	−1.632	−0.690	0.744	−0.610	−0.717	−0.838	23
S1	−1.849	0.118	0.493	−1.915	−0.157	−0.926	24

**Table A10:** Genetic diversity of 24 samples of *C. asiatica* in 14 SSR loci.

Locus	Na	Ne	Ho	He	H	I	PIC
SSR-1	6	5.67	0.04	0.96	0.82	1.76	0.80
SSR-2	4	3.79	0.17	0.83	0.74	1.36	0.69
SSR-3	3	2.48	0.57	0.43	0.60	1.00	0.53
SSR-6	3	2.79	0.05	0.95	0.64	1.06	0.57
SSR-7	3	2.91	0.58	0.42	0.66	1.08	0.58
SSR-8	5	4.05	0.17	0.83	0.75	1.49	0.71
SSR-10	3	2.62	0.83	0.17	0.62	1.02	0.54
SSR-11	4	2.95	0.25	0.75	0.66	1.22	0.61
mCaCIR006	4	3.48	0.13	0.88	0.71	1.29	0.66
mCaCIR007	4	2.91	0.09	0.91	0.66	1.21	0.61
mCaCIR027	3	2.71	0.13	0.87	0.63	1.04	0.56
mCaCIR028	3	2.25	0.21	0.79	0.55	0.94	0.49
mCaCIR029	3	2.66	0.24	0.76	0.62	1.03	0.55
mCaCIR030	4	2.83	0.00	1.00	0.65	1.13	0.58
Mean	3.71	3.15	0.25	0.76	0.67	1.19	0.61

Note: Na, number of alleles; Ne, effective number of alleles; Ho, observed heterozygosity; He, expected heterozygosity; H, *Nei’s* gene diversity index; I, Shannon’s information index; PIC, polymorphism information content.

**Table A11:** *Nei’s* genetic similarity coefficients and genetic distances for 24 *C. asiatica* samples.

	S1	S2	S3	S4	S5	S6	S7	S8	S9	S10	S11	S12	S13	S14	S15	S16	S17	S18	S19	S20	S21	S22	S23	S24
S1	**	0.86	0.79	0.52	0.50	0.63	0.67	0.53	0.64	0.68	0.51	0.49	0.52	0.51	0.57	0.50	0.53	0.60	0.56	0.51	0.55	0.57	0.58	0.52
S2	0.15	**	0.79	0.55	0.50	0.63	0.64	0.57	0.60	0.73	0.51	0.55	0.50	0.51	0.53	0.47	0.38	0.47	0.46	0.40	0.50	0.47	0.42	0.38
S3	0.24	0.23	**	0.55	0.54	0.69	0.64	0.50	0.67	0.54	0.58	0.53	0.52	0.61	0.50	0.64	0.53	0.48	0.53	0.52	0.60	0.48	0.47	0.44
S4	0.69	0.59	0.59	**	0.72	0.59	0.64	0.53	0.45	0.54	0.51	0.60	0.46	0.57	0.57	0.43	0.60	0.50	0.62	0.65	0.57	0.50	0.50	0.48
S5	0.84	0.84	0.76	0.37	**	0.69	0.63	0.41	0.53	0.57	0.41	0.44	0.41	0.56	0.79	0.59	0.54	0.43	0.54	0.66	0.57	0.51	0.43	0.43
S6	0.61	0.61	0.47	0.64	0.54	**	0.69	0.53	0.59	0.71	0.53	0.56	0.53	0.56	0.88	0.72	0.66	0.53	0.59	0.78	0.66	0.63	0.53	0.59
S7	0.40	0.44	0.44	0.44	0.56	0.47	**	0.76	0.82	0.76	0.77	0.75	0.71	0.61	0.63	0.64	0.64	0.69	0.60	0.65	0.52	0.69	0.62	0.69
S8	0.67	0.58	0.71	0.64	0.98	0.79	0.27	**	0.73	0.73	0.71	0.81	0.65	0.61	0.53	0.48	0.47	0.55	0.42	0.49	0.43	0.55	0.52	0.58
S9	0.46	0.50	0.39	0.77	0.72	0.64	0.20	0.32	**	0.76	0.81	0.89	0.88	0.71	0.67	0.67	0.53	0.55	0.44	0.51	0.45	0.58	0.54	0.59
S10	0.46	0.37	0.71	0.71	0.79	0.51	0.31	0.37	0.31	**	0.65	0.73	0.70	0.62	0.78	0.59	0.49	0.54	0.49	0.54	0.53	0.57	0.49	0.50
S11	0.71	0.67	0.54	0.67	0.98	0.79	0.26	0.35	0.21	0.49	**	0.85	0.88	0.76	0.47	0.71	0.62	0.64	0.53	0.56	0.51	0.58	0.62	0.65
S12	0.74	0.59	0.61	0.52	0.98	0.76	0.28	0.21	0.12	0.37	0.15	**	0.89	0.71	0.70	0.60	0.50	0.55	0.45	0.50	0.49	0.53	0.55	0.59
S13	0.67	0.69	0.64	0.77	0.98	0.76	0.33	0.42	0.13	0.39	0.12	0.11	**	0.72	0.60	0.69	0.59	0.62	0.53	0.49	0.51	0.52	0.59	0.63
S14	0.74	0.74	0.52	0.59	0.69	0.76	0.52	0.52	0.34	0.59	0.28	0.36	0.34	**	0.60	0.59	0.67	0.53	0.61	0.61	0.59	0.49	0.50	0.54
S15	0.65	0.68	0.80	0.65	0.30	0.19	0.52	0.68	0.43	0.30	0.84	0.40	0.55	0.62	**	0.67	0.57	0.50	0.50	0.70	0.57	0.60	0.50	0.57
S16	0.71	0.75	0.44	0.82	0.59	0.39	0.44	0.73	0.39	0.59	0.34	0.49	0.35	0.54	0.43	**	0.67	0.55	0.60	0.65	0.55	0.55	0.50	0.58
S17	0.69	0.99	0.64	0.52	0.69	0.51	0.46	0.79	0.66	0.85	0.51	0.69	0.54	0.43	0.65	0.40	**	0.88	0.95	0.81	0.79	0.71	0.78	0.88
S18	0.54	0.78	0.76	0.71	0.97	0.79	0.37	0.62	0.61	0.71	0.46	0.59	0.48	0.67	0.80	0.59	0.13	**	0.93	0.72	0.74	0.80	0.87	0.89
S19	0.60	0.77	0.62	0.49	0.69	0.64	0.50	0.86	0.79	0.85	0.63	0.79	0.63	0.52	0.80	0.50	0.06	0.07	**	0.78	0.83	0.75	0.79	0.82
S20	0.71	0.92	0.66	0.42	0.46	0.28	0.42	0.73	0.67	0.71	0.60	0.69	0.71	0.52	0.40	0.42	0.21	0.32	0.24	**	0.79	0.79	0.80	0.79
S21	0.65	0.74	0.55	0.58	0.73	0.57	0.67	0.87	0.78	0.84	0.69	0.77	0.69	0.60	0.73	0.60	0.24	0.30	0.19	0.24	**	0.66	0.82	0.73
S22	0.64	0.83	0.80	0.75	0.79	0.61	0.39	0.66	0.78	0.84	0.69	0.77	0.67	0.81	0.62	0.62	0.37	0.23	0.30	0.24	0.45	**	0.89	0.87
S23	0.61	0.92	0.79	0.73	0.97	0.79	0.51	0.71	0.57	0.69	0.57	0.65	0.54	0.77	0.80	0.73	0.26	0.14	0.24	0.23	0.21	0.12	**	0.92
S24	0.71	1.02	0.88	0.75	0.97	0.64	0.38	0.58	0.54	0.78	0.42	0.50	0.45	0.67	0.65	0.55	0.13	0.12	0.20	0.24	0.33	0.14	0.08	**

Note: Values above the diagonal represent *Nei’s* genetic similarity coefficients, whereas values below the diagonal represent *Nei’s* genetic distances. **, diagonal elements represent self-comparison.

**Table A12:** Similarity values of HPLC fingerprints for 24 *C. asiatica* accessions from Yunnan Province, China.

No.	Similarity	No.	Similarity	No.	Similarity
S1	0.959	S9	0.976	S17	0.988
S2	0.962	S10	0.973	S18	0.905
S3	0.938	S11	0.976	S19	0.943
S4	0.969	S12	0.884	S20	0.981
S5	0.907	S13	0.986	S21	0.917
S6	0.940	S14	0.964	S22	0.909
S7	0.966	S15	0.917	S23	0.918
S8	0.972	S16	0.959	S24	0.940

**Table A13:** Quantitative determination of five bioactive compounds in 24 *C. asiatica* accessions.

No.	Asiaticoside	Madecassoside	Asiaticacid	Madecassicacid	Kaempferol	Totalcontent
	(mg·g^−1^)	(mg·g^−1^)	(mg·g^−1^)	(mg·g^−1^)	(mg·g^−1^)	(mg·g^−1^)
S1	3.556	0.342	0.180	0.217	0.038	4.333
S2	4.494	0.963	0.363	0.310	0.067	6.197
S3	3.074	0.703	0.129	0.133	0.061	4.100
S4	13.93	0.611	0.318	0.130	0.069	15.059
S5	18.846	0.926	0.220	0.121	0.062	20.175
S6	3.490	0.602	0.083	0.106	0.083	4.364
S7	3.427	0.324	0.163	0.125	0.054	4.093
S8	4.442	0.521	0.442	0.292	0.066	5.763
S9	7.722	0.933	0.364	0.188	0.092	9.299
S10	3.822	1.044	0.183	0.173	0.057	5.279
S11	4.812	0.389	0.299	0.188	0.072	5.760
S12	6.057	1.209	0.278	0.328	0.043	7.915
S13	7.598	0.684	0.412	0.291	0.074	9.059
S14	4.825	1.412	0.257	0.302	0.103	6.899
S15	5.162	0.517	0.365	0.297	0.059	6.400
S16	5.669	1.389	0.681	0.203	0.087	8.029
S17	9.804	0.465	0.922	0.357	0.055	11.603
S18	8.926	0.358	0.267	0.111	0.041	9.703
S19	12.284	0.514	0.266	0.086	0.065	13.215
S20	5.709	0.855	0.574	0.408	0.045	7.591
S21	15.563	1.009	0.072	0.035	0.107	16.786
S22	9.484	0.453	0.301	0.040	0.040	10.318
S23	7.882	0.404	0.224	0.058	0.042	8.610
S24	6.251	0.335	0.390	0.116	0.046	7.138
Total content	176.829	16.962	7.753	4.615	1.528	207.687
Mean	7.368	0.707	0.323	0.192	0.064	8.654
Standard deviation	4.164	0.336	0.191	0.107	0.200	4.166
Minimum	3.074	0.324	0.072	0.035	0.038	4.093
Maximum	18.846	1.412	0.922	0.408	0.107	20.175
